# Genetic testing for fetal loss of heterozygosity using single nucleotide polymorphism array and whole-exome sequencing

**DOI:** 10.1038/s41598-024-52812-y

**Published:** 2024-01-25

**Authors:** Huili Xue, Aili Yu, Lin Zhang, Lingji Chen, Qun Guo, Min Lin, Na lin, Xuemei Chen, Liangpu Xu, Hailong Huang

**Affiliations:** 1 Medical Genetic Diagnosis and Therapy Center, Fujian Key Laboratory for Prenatal Diagnosis and Birth Defects, Fujian Maternity and Child Health Hospital, College of Clinical Medicine for Obstetrics & Gynecology and Pediatrics, Fujian Medical University, No. 18 Daoshan Road, Gulou District, Fuzhou City, 350001 Fujian Province China; 2 Reproductive Medicine Center, Fujian Maternity and Child Health Hospital College of Clinical Medicine for Obstetrics & Gynecology and Pediatrics, Fujian Medical University, No. 18 Daoshan Road, Gulou District, Fuzhou City, 350001 Fujian Province China; 3 Fujian Medical University, No. 88 Jiaotong Road, Cangshan District, Fuzhou City, 350001 Fujian Province China

**Keywords:** Genetics, Medical research, Molecular medicine

## Abstract

The study explored the clinical significance of fetal loss of heterozygosity (LOH) identified by single-nucleotide polymorphism array (SNP array). We retrospectively reviewed data from pregnant women who underwent invasive diagnostic procedures at prenatal diagnosis centers in southeastern China from December 2016 to December 2021. SNP array was performed by the Affymetrix CytoScan 750 K array platform. Fetuses with LOH were further identified by parental verification, MS-MLPA, and/or trio whole-exome sequencing (trio-WES). The genetic results, fetal clinical manifestations, and perinatal outcome were analyzed. Of 11,062 fetuses, 106 (0.96%) had LOH exhibiting a neutral copy number, 88 (83.0%) had LOH in a single chromosome, whereas 18 (17.0%) had multiple LOHs on different chromosomes. Sixty-six fetuses had ultrasound anomalies (UAs), most frequently fetal growth restriction (18/66 (27.3%)). Parental SNP array verification was performed in 21 cases and trio-WES in 21 cases. Twelve cases had clinically relevant uniparental disomy, five had pathogenic variants, four had likely pathogenic variants, six had variants of unknown significance, and eight had identity by descent. The rate of adverse pregnancy outcomes in fetuses with LOH and UAs (24/66 (36.4%)) was higher than in those without UAs (6/40 (15.0%)) (*p* < 0.05). LOH is not uncommon. Molecular genetic testing techniques, including parental SNP array verification, trio-WES, methylation-specific multiplex ligation-dependent probe amplification, regular and systematic ultrasonic monitoring, and placental study, can accurately assess the prognosis and guide the management of the affected pregnancy.

## Introduction

In prenatal diagnostics, chromosomal microarray analysis (CMA) improved the diagnostic rate for chromosomal abnormalities by 4.1–10% compared with traditional karyotyping in fetuses with ultrasound anomalies (UA). Single-nucleotide polymorphism array technology can not only identify copy number variation (CNV) but also detect chromosome aneuploidy and haploidy, triploidy, loss of heterozygosity (LOH), uniparental disomy (UPD), and low-level mosaicism^1,2^.

LOH, referred to as the region of homozygosity in a chromosome, and the clinical implications, are associated concerns for identity by descent (IBD), consanguinity, UPD, and recessive single-gene mutations. LOH confirmed to have been inherited from only one parent is called UPD, which can lead to imprinting disorders involving imprinted chromosomes 6, 7, 11, 14, 15, and 20. Moreover, UPDs are classified as either isodisomy, heterodisomy, or mixed UPD, according to the parental origin. Common mechanisms resulting in UPD include trisomy rescue, monosomy rescue, and somatic mitotic recombination, resulting in mosaic segmental UPD^3,4^.

Assessing the prognosis of fetuses with LOH in prenatal diagnosis is challenging. Thus, understanding its clinical significance is necessary due to the phenotypic complexity of LOH and its uncertain pathogenicity. To assess the clinical significance and better understand the correlation between LOH and its phenotype, we investigated the clinical manifestations; performed further molecular genetic analysis using parental SNP array verification, trio whole-exome sequencing (WES), and methylation-specific multiplex ligation-dependent probe amplification (MS-MLPA); and tracked the perinatal outcomes of fetuses with LOH.

## Patients and methods

### Subjects

We retrospectively reviewed pregnant women undergoing invasive diagnostic procedure for a variety of indications at all prenatal diagnosis centers in Fujian Province in southeastern China, from December 2016 to December 2021. Most of the cases came from Fujian Maternity and Child Health Hospital. Obtaining informed consent from all the pregnant couples, fetal samples were collected via invasive diagnostic procedure according different weeks of gestation. The study was approved by the Ethics Committee of the Fujian Maternity and Child Health Hospital (No.2016KYLLD01051).

### Conventional karyotyping analysis

Karyotyping was performed following the standard cytogenetic protocol^5^, and karyotypes were scanned on Leica GSL120. At least 20 metaphases were counted, and five metaphases were analyzed. Karyotype analysis and description were based on ISCN 2020^6^.

### Isolation of genomic DNA

Fetal sample, 15 mg of chorionic villi, 30–40 mL of amniotic fluid, or 2–5 mL umbilical cord blood was obtained, and genomic DNA from the fetus and its parents were extracted using the QIAamp® DNA Blood Mini Kit (Qiagen Inc., Hilden, Germany) following the manufacturer’s instructions, and maternal cell contamination was ruled out using multiplex quantitative-fluorescent polymerase chain reaction Darui kit (Darui, Guangzhou, China), which was tested on 20 markers including: four short tandem repeats (STRs) from chromosome 13 (D13S634, D13S305, D13S628, D13S742), four from chromosome 18 (D18S391, D18S1002, D18S535, D18S386), six from chromosome 21 (D21S1411, D21S1445, D21S1414, D21S1412, D21S1433, 21q11.2), and six STRs from chromosome X and Y (AMXY, DXS1187, DXS8377, SRY, DXS6809, DXS981).

### Single nucleotide polymorphism array and data analysis

Chromosomal aberrations, CNVs and LOH were detected using a SNP array on a CytoScan 750 K (Affymetrix Inc., Santa Clara, CA) platform, all the experimental processes of SNP array were performed as previously described^7^. Parental SNP array verification was performed to confirm the origin of fetal LOH.

The raw data were analyzed using the Affymetrix Chromosome Analysis Suite software (version 3.1.0.15). The coordinate of the chromosome was described based on the genome version hg19. CNVs were classified according to the American College of Medical Genetics (ACMG) guidelines^8^. The reporting threshold was set at CNV ≥ 500 Kb, and LOHs cutoffs of > 5 Mb for telomeric LOH and/or > 15 Mb for interstitial LOH on imprinted chromosomes (chromosome 6, 7, 11, 14, 15, and 20); > 10 Mb for telomeric LOH and/or > 20 Mb for interstitial LOH on nonimprinted chromosomes; > 15 Mb for interstitial LOH on imprinted chromosomes or 20 Mb for an interstitial LOH on nonimprinted chromosomes; percentage of LOH was larger than or equal to 1.625%^9^. The sex chromosomes are excluded, because males have one X and one Y chromosome and therefore cannot have LOH at any site apart from the pseudoautosomal regions^10^. The significance of LOH results was interpreted through PubMed (http://www.ncbi.nlm.nih.gov/pubmed), Online Mendelian Inheritance in Man (OMIM; http://www.omim.org/), DECIPHER (https://decipher.sanger.ac.uk/), UCSC (http://genome.ucsc.edu/), ClinGen Dosage Sensitivity Map (https://www.ncbi.nlm.nih.gov/projects/dbvar/clingen/index.shtml), uniparental disomy (http://cs-tl.de/DB/CA/UPD/0-Start.html), Geneimprint (http://www.geneimprint.com/), the Catalogue of Imprinted Genes (www.otago.ac.nz/IGC), the Human Gene Mutation Database (http://www.hgmd.cf.ac.uk/ac/index.php), the Locus-Specific Mutation Database (http://www.hgvs.org/dblist/glsdb.htm) and the 1000 Genomes Project Dataset (https://www.ncbi.nlm .nih.gov/variation/tools/1000genomes/).

### WES and bioinformatics analysis

To identify homozygous mutations of recessive diseases in addition to UPD, trio-WES (for fetuses and parents) was carried out with the informed consent of pregnant couples. After the sample genomic DNA was extracted, exons were captured using Agilent Sure Select Technology (Agilent, Santa Clara, CA, USA), fragmented randomly, purified, and enriched to construct DNA libraries. Paired-end sequencing was performed on Illumina HiSeq 2500 (Illumina, USA) instruments according to the manufacturer’s instructions (Illumina, San Diego, CA, USA).

For sequence alignment, variant calling, and annotation, the sequences were mapped to their locations with the human genome reference sequence (hg19 build) using Burrows-Wheeler software (version 0.59)^11^. All SNVs and InDels were annotated with public population frequency databases, including NCBI dbSNP, 1000 Genomes Project, the Exome Aggregation Consortium, as well as OMIM, Swiss-var, Human Gene Mutation Database, ClinVar, and other disease databases, and only variants that were clinically or potentially relevant to the patients’ phenotype were reported. Mutations were annotated, protein function effects and shear harmfulness were predicted, and the pathogenicity of the variants was assessed according to ACMG^12^.

### MS-MLPA

Methylation analysis of 7q21.13q36.3 loci was performed using MS-MLPA (SALSA MS-MLPA probe mix ME030-C3 BWS/RSS; MRC Holland, Amsterdam, The Netherlands), and the relative copy numbers of the three methylation probes in the *MEST* gene (maternal methylation gene region, with paternal methylation preferentially expressed) on chromosome 7q32.2 was determined. All procedures were performed following the manufacturers’ protocols as previously described^13^.

### Statistical analysis

In fetuses with LOH, we collected data relating to basic information, imaging findings, serological Down's screening results, non-invasive prenatal testing results, invasive diagnostic testing results, further genetic analysis, perinatal outcomes, and follow-up information. Perinatal outcomes in our hospital were obtained from delivery records. Data relating to cases from other centers were followed up via telephone.

### Ethics approval and consent to participate

SPSS software version 22.0 was used for statistical analysis. Measurement data were expressed as mean ± standard deviation, statistical comparisons were performed using χ^2^ test, and *p* < 0.05 was considered statistically significant.

### Ethics approval and consent to participate

The study complied with the principles set forth in the Declaration of Helsinki. It was approved by the Institutional Review Board of Fujian Maternal and Child Health Hospital. Written informed consent was obtained from each patient.

## Results

### Patient characteristics

In total, 11,062 fetuses undergoing invasive diagnostic testing over a period of five years were analyzed using SNP array, and 106 fetuses had LOH. The detection rates of fetal LOH for different invasive diagnostic indications are shown in Table 1. The mean weeks of gestation at invasive prenatal diagnosis and maternal age for pregnancies with fetal LOH were 21 ± 1 (range, 11^+6^ to 31) and 31 ± 3 (range, 19–42) years, respectively. The detailed parental SNP array verification results, trio-WES results, MS-MLPA results, ultrasound findings, and perinatal outcomes of 42 fetuses with LOH are summarized in Table 2, and the data regarding the remaining 64 prenatally diagnosed cases with LOH that declined further genetic testing are listed in Table 3.

**Table 1:** The detection rate of fatal LOH by SNP array for different invasive testing indications.

Indication for invasive testing N	Case number n (%)	Fetal LOH detected
Ultrasonic structural anomalies	2749	23 (0.84)
Other ultrasound anomalies	4830	47 (0.97)
Positive NIPT result	1462	10 (0.68)
High risk for Down’s screening	376	6 (1.86)
Parental abnormal karyotype	385	1 (0.26)
Consanguinity	15	6 (6/15*)
Previous adverse pregnancy	705	3 (0.43)
Advanced maternal age	540	14 (2.59)
Total	11,062	106 (0.96)

Data are given as *n* (%).

Each case was classified based on most important indication. Classification of indications arranged from most to least important was as follows: ultrasound structural anomalies, fetal karyotype abnormality, soft marker, positive NIPT results, high risk for Down’s screening, parental genetic factors, consanguinity, previous adverse pregnancy, AMA and others.

*If cases number is less than 20, the percentage is not calculated.

^#^Other ultrasound anomalies included soft-marker anomalies, FGR or fetal overgrowth, hydramnios, hydramnios and abnormal blood flow on doppler ultrasound.

*LOH*, loss of heterozygosity; *NIPT*, non-invasive prenatal testing; *SNP*, single nucleotide polymorphism.

**Table 2:** Forty-two fetuses with LOH accepted further genetic testing.

Case	GW	Prenatal ultrasound findings/Invasive testing indication§	Fetal karyotype	Fetal LOH region (hg 19) detected by SNP-array	Size (Mb)/Percentage (%)*	Results of further genetic testing (classification of pathogenicity)	Outcome
1^a^	22	Oligohydramnios, strephenopodia, consanguineous marriage	46, XX	1p36.33p36.21(888,658–14,505,595) × 2 hmz 2q11.1q14.3(95,550,957–127,459,321) × 2 hmz	13.6 Mb 31.9 Mb (3.69%)	Prenatal trio-WES indicated a homozygous mutation, NM_000302: c.2071_2072 ins CC (p.R693Qfs*122), in *PLOD1* (153,454) in the fetus, associated with Ehlers-Danlos syndrome type 1 (OMIM 225,400) which was located in the LOH of 1p36.33p36.21, and both parents were heterozygous for this variation (LP)	Term birth, neonatal death
2	21	Decreased fetal FL/BPD and FL/HC ratio	46, XY	12q23.3q24.32(106,008,417–126,307,790) × 2 hmz	20.3 Mb	Postnatal trio-WES indicated a heterozygous mutation, NM_001356: c.45 + 1G > C, in *DDX3X*, which is associated with intellectual developmental disorder, X-linked, syndrome, Snijders Blok type (MRXSSB, OMIM 300,958) (P)	Preterm birth at 36 weeks, BW 2.75 kg diagnosed with hand deformity, autism
3	19 +	FGR?intracardiac echogenic focus, increased umbilical artery resistance index, oligohydramnios	46, XX	6p25.3p23(203,878–13,411,320) × 2 hmz 6p21.1p11.1(41,305,454–58,726,706) × 2 hmz 6q11.1q14.1(61,972,918–75,972,465) × 2 hmz 6q22.31q25.1(123,041,062–149,830,858) × 2 hmz (containing imprinted genes *PLAGL1* (603,044) and *HYMAI* (606,546))	13.2 Mb 17.4 Mb 13.9 Mb 26.7 Mb	Prenatal trio-WES indicated mix UPD (6) mat (P)	TOP
4	26 + 3	Polyhydramnios (AFI 38.7 cm)	46, XY	arr (14) × 2 mos hmz (80%)	Mosaicism 80%	Parental SNP array verification indicated UPD (14) pat, which is associated with Kagami-Ogata syndrome (P)	TOP
5	24	Multiple malformations, thickened prenasal skin, "fish mouth" shape mouth	46, XX	1p36.11p21.2(26,044,678–99,969,487) × 2 hmz 3p25.3p14.2(8,746,546–60,897,120) × 2 hmz 6p25.1p22.1(5,596,983–28,449,315) × 2 hmz 6q24.2q25.2(143,241,806–155,371,652) × 2 hmz 7p14.3p11.1(33,991,108–58,019,983) × 2 hmz 7q11.21q22.1(62,569,501–98,898,149) × 2 hmz 11p15.1p11.12(17,372,347–51,550,787) × 2 hmz 11q11q14.1(54,827,207–84,312,637) × 2 hmz	73.9 Mb 60 Mb 22.9 Mb 12.1 Mb 22.8 Mb 24 Mb 34.2 Mb 29.5 Mb (10%)	Postnatal trio-WES indicated a homozygous mutation, NM_173076.3: c.6577_657 del, in *ABCA12* in the fetus, associated with autosomal recessive congenital ichthyosis type 4B type (OMIM 242,500) and 4A type (OMIM 601,277) (P)	Term birth, BW 3.2 kg The collodion female baby died five days after birth
6	13^+6^	FGR	46, XY	7q31.31q36.3(120138084_159118443) × 2 hmz	39 Mb	Parental SNP array verification indicated UPD (7) mat, which is associated with Silver-Russell syndrome (P)	TOP
7	18^+^	AMA	46, XX	2q22.3q31.1(144,624,648–173,171,481) × 2 hmz 4q13.1q21.3(63,730,113–87,252,596) × 2 hmz	28.5 Mb 23.5 Mb	Prenatal trio-WES indicated no clinically relevant mutations	Term birth (Normal phenotype)
8	23^+^	Small fetal BPD and HC for gestation age	46, XX	14q32.11q32.31(91556694_101593701) × 2 hmz	10 Mb	Prenatal trio-WES indicated no clinically relevant mutations	Term birth (Normal phenotype)
9	24	Fetal ARSA	46, XY	1p36.21p35.2(15,728,288–31,781,279) × 2 hmz 4p15.2p11(25,981,952–49,063,479) × 2 hmz	16 Mb 23 Mb	Prenatal trio-WES indicated no clinically relevant mutations., and identified a homozygous mutation, NM_015378: c.427A > G (p.R143G), in *VPS13D* in the fetus, associated with autosomal recessive spinocerebellar, ataxia, autosomal recessive 4 (SCAR4, OMIM 607,317) (VOUS)	Term birth (Normal phenotype)
10	25	Fetal bilateral renal enlargement, increased renal echogenicity	46, XY	8q11.23q24.3(55365228_146292734) × 2 hmz	90.9 Mb	Prenatal trio-WES indicated UPD (8q11.23-q24.3) mat (VOUS)	Term birth (Normal phenotype)
11	26	Small fetal BPD was small for gestation age, consanguineous marriage	46, XX	14q13.1q24.2(34435418_72618432) × 2 hmz	38 Mb	Postnatal trio-WES confirmed no indication of UPD (14), and showed a heterozygous mutation, NM _000095: c.1417_1419 dup (p. D473dup), in *COMP* in the female infant, associated with autosomal dominant pesudoachondroplasia (PSACH, OMIM 177,170), epiphyseal dysplasia, multiple, 1 (EDM1, OMIM 132,400), and carpal tunnel syndrome 2 (CTS2, OMIM 619,161) (LP)	Term birth Now the infant is 5 months old, 60 cm tall, and often arches her back, Brain MRI revealed that bilateral ventricles were asymmetrical, and the left lateral ventricle was larger than the right. Some of the extracerebral spaces are slightly widened. Low T1W1 and high T2W1 signals were observed in bilateral maxillary sinus, ethmoid sinus and sphenoid sinus
12	22	FGR; high risk of trisomy 15 detected by NIPT; AMA	46, XY	15q21.3q26.1(53188649_90583138) × 2 hmz	37.4 Mb	Prenatal trio-WES indicated mixed UPD (15q21.3q26.1) mat associated with PWS (P)	TOP
13	18	Thickened nuchal translucency (2.8 mm), cervical lymphatic hygroma	46, XX	6p12.2p11.1(52607147_58726706) × 2 hmz 6q11.1q12(60972918_66615551) × 2 hmz	6.1 Mb 5.6 Mb	Prenatal trio-WES indicated no clinically relevant mutations	TOP
14	25	Fetal RAA-ARSA	46, XY	7q21.13q36.3(88,712,610–159,118,443) × 2 mos hmz (45%)	70.4 Mb	First, parental SNP array verification indicated it is not possible to determine whether the LOH of chromosome 7 is paternal or maternal; Then, MS-MLPA for the methylation analysis of 7q21.13q36.1 loci, MS-MLPA revealed that the relative copy numbers of the three methylation probes (184 bp, 190 bp, and 256 bp) in *MEST* gene (maternal methylation gene region, paternal methylation was preferentially expressed) were 0.66, 0.66 and 0.64, respectively, suggesting a possible low proportion mosaic UPD (7) mat. However, the experimental results are near the threshold range, so it cannot be interpreted accurately (P)	Term birth Feeding difficulties exist after birth, now 3 months after delivery, the child's height and development is normal, only light weight
15	23 +	Fetal bilateral renal enlargement, increased renal echogenicity	46, XX	6p12.3q14.1(46587519_77260358) × 2 hmz	27.45 Mb (0.95%)	Prenatal trio-WES revealed a homozygous mutation, NM_138694.4: c.1233G > A p.K411K, in *PKHD1* in the fetus, associated with autosomal recessive polysystic kidney disease 4, with or without hepatic Disease (OMIM 263,200) (P)	TOP
16	21 +	Reproductive history of children with chromosomal abnormalities (arr[hg19]9p24.3p22.3(203861_16540793) × 1, 9p22.3p13.3(16540940_34942483) × 3, which is associated with 9p partial monosomy syndrome and 9p trisomy, respectively; AMA	46, XY	18p11.32p11.21(136305_15079294) × 2 hmz 18q11.1q23(18552517_77997606) × 2 hmz	15 Mb 59 Mb	Prenatal trio-WES indicated UPD (18) pat, and also revealed a heterozygous splicing mutation, NM_006767.4: c.2069 + 1G > A, in *LZTR1* in the fetus, associated with noonan syndrome 2 (NS10, OMIM 616,564) and schwannomatosis-1, susceptibility, (OMIM 615,670), inherited paternally (LP)	Term birth
17	18 +	NIPT indicated a 8.2 Mb deletion at 4q31-qter (FF: 6.4%)	46, XY	4q32.3q35.2(167230247_190921709) × 2 hmz	23.7 Mb	Trio-WES indicated iso UPD (4q32.3q35.2) mat [(UPD (4)], and also revealed a heterozygous splicing mutation, NM_001807.6, in *CEL* in the fetus, associated with maturity-onset diabetes of the young, type VIII (OMIM 609,812), inherited paternally (LP)	Term birth. Currently developing normally at 2 years old
18	20	Lethal bone dysplasia (osteogenesis imperfecta type II)	46, XX	5q23.2q32(125,771,613–145,714,232) × 2 hmz	20 Mb	Prenatal trio-WES indicated a heterozygous de novo missense variant, NM_000088: c.1436G > C p.G479A, in *COL1A1* (120,150) on chromosome 17, associated with osteogenesis imperfecta, type I (OMIM 166,200), type II (OMIM 166,210), type III (OMIM 259,420), type IV (OMIM 166,220), Ehlers-Danlos syndrome, arthrochalasia type 1 (OMIM 130,060), Caffey disease (OMIM 114,000), and bone mineral density variation QTL, steoporosis (OMIM 166,710) (P)	TOP
19	28 +	FGR	46, XX	15q14q21.3(35,077,111–54,347,324) × 2 hmz	19.2 Mb	Parental SNP array verification indicated UPD (15) mat, associated with PWS (P)	TOP
20	27 +	Narrow inner diameter of aortic arch, increased renal echogenicity, FGR, persistent left superior vena cava, thickening of the placenta	46, XY	2p25.3p11.2(50,813–87,053,152) × 2 hmz 2q11.1q37.3(95,550,957–242,773,583) × 2 hmz	82.0 Mb 147.2 Mb	Parental SNP array verification indicated UPD (2) mat (VOUS)	TOP
21	23 +	VSD, FGR, aortic stenosis, left kidney dysplasia or absence, enhanced intestinal echo	46, XX	16q23.2q24.3(79,800,878–90,146,366) × 2 hmz 16p13.3p12.3(94,807–19,302,326) × 2 hmz	10.3Mb 19.2Mb	Parental SNP array verification indicated UPD (16) mat (P)	TOP
22	22 +	FGR, mild tricuspid regurgitation, reverse a-wave of ductus venosus, enhanced intestinal echo	46, XY	6p25.3q27(203,877–170,896,644) × 2 hmz	170.7 Mb	Parental SNP array verification indicated UPD (6) pat, associated with transient neonatal diabetes (P)	TOP
23	19	Amniocentesis: 47, XX, + mar dn; high risk of trisomy18 screening	47, XX, + mar dn	3p12.1p11.1(85,527,865–90,130,204) × 3, 3q11.1q11.2(93,674,112–95,321,299) × 3 3p13p12.1(72,069,483–86,073,653) × 2 hmz 3q11.2q22.3(95,320,713–137,634,506) × 2 hmz	14.0 Mb 42.3 Mb	Parental SNP array verification indicated UPD (3) mat (VOUS)	Term birth, currently developing normally at 1.5 years old
24	29	Sever FGR	46, XX	15q11.2q22.2(22,817,870–62,568,746) × 2 hmz 15q26.2q26.3(96,143,558–102,397,317) × 2 hmz	39.7 Mb 6.25 Mb	Parental SNP array verification indicated UPD (15) mat, associated with PWS (P)	TOP
25		FGR, high risk of trisomy 21 screening	46, XY	11p15.5p15.4(205,827–8,150,933) × 2 hmz	8 Mb	Parental SNP array verification indicated UPD (11) mat, associated with Silver-Russell syndrome (P)	TOP
26	30	Severe FGR	46, XX	7p22.3p12.2(50944_157155880) × 2 hmz	50.4 Mb	Parental SNP array verification indicated UPD (7p22.3p12.2) mat, associated with Silver-Russell syndrome (P)	TOP
27	23	Fetal HC was less than the mean 3SD; high risk of trisomy 16 detected by NIPT (Z = 18.705)	46, XX	16q22.3q24.3(73705779_90146366) × 2 hmz	16 Mb	Parental SNP array verification indicated mixed UPD (16) mat (VOUS)	TOP
28	25	FGR; high risk of trisomy 16 detected by NIPT (Z = 25.2); AMA,	46, XY	6p25.3q27(203,877–170,896,644) × 2 hmz	170.7 Mb	Parental SNP array verification indicated mixed UPD (6) mat (VOUS)	TOP
29	23	Fetal bilateral femoral curvature, less than the normal predictive value -2SD	46, XY	5p15.33p11(113,576–46,242,541) × 2 hmz	46.1 Mb	Prenatal trio-WES indicated an inherited paternally splicing variant, NM_000088.4: c.1615-1G > T (p. G802V), in *COL1A1* (120,150) on chromosome 17, associated with autosomal dominant osteogenesis imperfecta (P)	TOP
30	29	Hydrops fetalis, FGR	46, X, i(Xq) [40]/45, X [14]	Xp22.33p11.22(168,551–54,572,730) × 1 11p15.5p15.4(230,750–8,050,928) × 2 hmz	7 Mb	Parental SNP array verification indicated UPD (11) mat, associated with Silver-Russell syndrome (P)	TOP
31	26	FGR, high risk of trisomy 21 screening	46, XX	7q11.21q11.23(62569502_75233244) × 2 hmz 7p14.1p11.1(42421781_58019983) × 2 hmz 9q21.13q22.2(78780578_92509057) × 2 hmz	12.7 Mb 15.6 Mb 13,7 Mb	Prenatal trio-WES indicated no clinically relevant mutations	Term birth, currently developing normally at 2 years old
32	18	High risk of trisomy 21 screening	46, XY	7q22.3q31.32(107306055_122301501) × 2 hmz	15 Mb	Prenatal trio-WES indicated no clinically relevant mutations	Term birth, currently developing normally at 2.5years old
33	19	Deep notch a-wave of ductus venosus	46, XX	13q14.2q21.2(47358540_61986828) × 2 hmz	14.6 Mb	Prenatal trio-WES indicated no clinically relevant mutations	Term birth, currently developing normally at 2 years old
34	25	Fetal micrognathia, small BPD, HC and FL for gestation age	46, XY	5q15q22.1(94849287_110237725) × 2 hmz	15.39 Mb	Prenatal trio-WES indicated no clinically relevant mutations	Term birth, micrognathia
35	23	Fetal suprachivalal stenosis	46, XX	1p31.3p31.1(65532190_78558941) × 2 hmz	13.03 Mb	Prenatal trio-WES indicated no clinically relevant mutations	Term birth, normal development
36	22	Fetal pulmonary sequestration	46, XX	2p24.3p21(16,435,384–42,306,722) × 2 hmz	25.8 Mb	Parental SNP array verification indicated IBD	Term birth, normal development
37	18	Thickened nuchal translucency (3.5 mm); AMA	46, XY	14q11.2q12(22,094,953–32,626,332) × 2 hmz	10.5 Mb	Parental SNP array verification indicated IBD	Term birth, normal development
38	19	Pregnant women had a child with mental retardation; AMA	46, XX	3p22.1p14.2(42390369_60178833) × 2 hmz	17.7 Mb	Parental SNP array verification indicated IBD	Term birth, normal development
39	18	Previous adverse pregnancy	46, XY	15q21.2q22.2(49971494_63093257) × 2 hmz	13.1 Mb	Parental SNP array verification indicated IBD	Term birth, normal development
40	18 +	FGR, previous adverse pregnancy	46, XY	6p21.31p11.1(33808094_58726706) × 2 hmz 6q11.1q16.1(61972918_93717031) × 2 hmz	24.9 Mb 31.7 Mb	Parental SNP array verification indicated IBD	Term birth, normal development
41	19	AMA	46, XX	6q14.3q21(87,299,268–110,741,585) × 2 hmz	23.4 Mb	Parental SNP array verification indicated IBD	Term birth, normal development
42	20	Thickened nuchal translucency (3.0 mm); AMA	46, XX	1q21.1q21.2(144,077,593–148,750,533) × 2 hmz 3p21.31p21.1(48,166,782–53,172,233) × 2 hmz 5q21.3q22.1(107,196,975–110,478,806) × 2 hmz 12q21.31q21.33(82,446,525–91,707,400) × 2 hmz 14q31.2q32.12(84,339,970–92,755,472) × 2 hmz 15q24.1q25.3(73,065,223–87,467,262) × 2 hmz 16p13.3(94,807–3,112,982) × 2 hmz 17p12p11.2(15,838,698–22,170,994) × 2 hmz	54.2 Mb (2%)	Parental SNP array verification indicated IBD	Term birth, normal development

*The percentage is calculated by the sum of the size (Mb) of the LOH segments over 5 Mb divided by ~ 2781 Mb.

^**§**^Each case was classified based on its most important invasive diagnostic indication. The classification of indications, arranged in the order of importance are as follows: ultrasound anomalies, positive NIPT results, high risk for Down’ screening, parental abnormal karyotype, consanguinity, previous adverse pregnancies, AMA, suspected fetal infection, and others.

*AMA*, advanced maternal age; *AFI*, amniotic fluid index; *ARSA*, aberrant left subclavian artery; *BW*, birth weight; *BPD*, biparietal diameter; *FL*, femur length; *FGR*, fetal growth restriction; *GW*, gestational weeks; *HC*, head circumference; *IBD*, identity by descent; *LOH*, loss of heterozygosity; *LP*, likely pathogenic; *Mat*, maternal; *MS-MLPA*, methylation-specific multiplex ligation-dependent probe amplification; *NIPT*, noninvasive prenatal testing; *OMIM*, online mendelian inheritance in man; *Pat*, paternal; *P*, pathogenic; *PWS*, Prader-Willi syndrome; *RAA*, right aortic arch; *SNP*, single nucleotide polymorphism; *TOP*, terminal of pregnancy; *UPD*, uniparental disomy; *VSD*, ventricular septal defect.

**Table 3:** The detailed 64 prenatally diagnosed fetuses with LOH declined parental SNP array verification or further genetic testing.

Case	GW	Prenatal imaging findings/Invasive testing indication	Fetal karyotype	Fetal LOH region detected by SNP-array results [hg19]	Size (Mb)/Percentage (%)*	Outcome
43	18 +	The husband carries 46, XY, t (4;5) (q31; q23), consanguineous marriage	46, XX, t (4;5) (q31; q23) pat	18p11.32p11.21(136,304–15,079,294) × 2 hmz	15 Mb	Term birth, normal development
44	25	FGR, enhanced intestinal echo	46, XX	8p23.1p21.3(8117565_21609098) × 2 hmz	13.5 Mb	Term birth, normal development
45	23 +	Small femur length for gestation age; high risk of trisomy 22 detected by NIPT	46, XY	22q12.3q13.33(35,156,334–51,157,531) × 2 hmz	16 Mb	Term birth, normal development
46	20	Del (8p) detected by NIPT; AMA	46, XY	8p23.3p23.1(168,483–6,999,220) × 2 hmz, 8p23.1p12(8,117,564–32,069,805) × 2 hmz	6.8 Mb 23.9 Mb	Term birth, normal development
47	18 +	Consanguineous marriage; pregnant women had a child with mental retardation	46, XX	8p23.3p23.1(2,142,556–6,999,220) × 2 hmz	67.9 Mb (3.63%)	Term birth, mental retardation
48	21	Enhanced intestinal echo; Pregnant women had three children with cardiac malformation	46, XX	13q14.2q22.3(49,157,476–78,073,267) × 2 hmz	29 Mb	Term birth, normal development
49	12	Fetal cystic hygroma	46, XY	9q21.13q21.33(77,263,747–89,236,178) × 2 hmz 9q22.33q31.1(101,366,537–107,749,439) × 2 hmz 10q22.3q24.1(80,035,256–97,660,572) × 2 hmz 14q31.1q32.2(79,435,542–97,071,550) × 2 hmz 17q24.2q25.3(67,002,309–81,041,760) × 2 hmz 18q22.3q23(71,555,375–77,997,606) × 2 hmz 19q12q13.33(31,360,714–49,513,502) × 2 hmz 21q21.1q22.13(19,573,963–39,057,622) × 2 hmz	111.74 Mb (4.02%)	Missed abortion
50	14 +	AMA	46, XY	9q22.33q33.1(101944435_121284276) × 2 hmz	19.3 Mb	Missed abortion
51	20	Coarctation of the fetal aorta	46, XX	19p13.2p11(6939202_24462369) × 2 hmz	17.5 Mb	Intrauterine demise
52	15	Hydrops fetalis	46, XY	5q11.1q35.3(49564628_180692321) × 2 mos hmz (30%)	130 Mb	Missed abortion
53	16	Fetal giant bladder	46, XX	5q34q35.3(164130490_176629546) × 2 hmz	12.5 Mb	Missed abortion
54	25	Fetal dysplasia or absence of left radius, abnormal left lower limb posture, single umbilical artery	46, XY	16q22.3q24.3(73294159_90146366) × 2 hmz	16.8 Mb	TOP
55	15	Thickened NT (5 mm)	46, XX	3p14.2p11.1(59365038_90485635) × 2 hmz 3q11.1q13.11(93558926_103243507) × 2 hmz	31 Mb 10 Mb	Missed abortion
56	23	Small femur length and humerus length for gestation age	46, XX	4q28.3q31.3(133,718,289–154,569,367) × 2 hmz	20.8 Mb	Term birth, normal development
57	26 +	Fetal ventriculomegaly (1.4 cm), large for gestational age	46, XY	3q26.1q29(163,256,369–197,791,601) × 2 hmz 5p13.1p11(41,029,137–46,313,469) × 2 hmz 6q24.2q26(143,341,406–161,527,784) × 2 hmz 12q13.2q21.2(56,011,100–77,134,151) × 2 hmz 17q21.2q21.32(39,639,602–45,479,706) × 2 hmz 21q21.3q22.2(28,124,165–42,352,287) × 2 hmz	99.1Mb (3.57%)	TOP
58	24	Fetal infantile polycystic kidney disease?	46, XX	2q31.1q35(175,428,638–216,907,322) × 2 hmz, 5q14.3q22.3(85,447,858–113,850,052) × 2 hmz	41 Mb 28 Mb	TOP
59	28 +	FGR, small fetal HC for gestation age	46, XY	5q15q22.2(94637916_112744413) × 2 hmz	18.1 Mb	Preterm birth
60	19	AMA	46, XY	10q23.1q25.1(86,546,612–109,956,967) × 2 hmz	23.4 Mb	Term birth, normal development
61	25	Fetal tetralogy of Fallot, thickened NT (4.7 mm)	46, XY	1p33p32.3(50,051,514–53,274,566) × 2 hmz 2q23.3q24.1(153,771,280–158,783,675) × 2 hmz 3q21.2q22.1(124,817,983–129,317,745) × 2 hmz 3q22.1q23(133,262,566–139,418,898) × 2 hmz 3q26.1q26.2(161,540,639–168,592,236) × 2 hmz 7p22.3p21.2(2,707,568–13,857,235) × 2 hmz 8q23.3q24.12(114,788,423–119,897,611) × 2 hmz 9p22.1p13.3(19,696,747–36,125,149) × 2 hmz 11q12.2q12.3(60,193,879–63,210,491) × 2 hmz 11p11.2p11.12(45,781,075–51,550,787) × 2 hmz 17q21.31q21.32(41,647,165–44,927,874) × 2 hmz 17q25.1q25.3(71,965,953–75,785,426) × 2 hmz 20p11.23p11.21(20,268,153–23,275,237) × 2 hmz	77.5 Mb (2.8%)	TOP
62	23 +	AMA	46, XY, t (11;22) (q24.1; q12.3) pat	3p13q13.31(71,435,373–116,447,779) × 2 hmz	45.0 Mb	Term birth, normal development
63	22	Fetal diaphragmatic hernia, permanent right umbilical vein, high risk of trisomy 21 screening	46, XY	22q11.1q13.32(16,888,899–48,538,372) × 2 hmz	31.6 Mb	TOP
64	19 +	High risk of trisomy 21 screening, consanguineous marriage	46, XX	8q23.1q23.3(109,495,878–115,007,395) × 2 hmz 8q24.21q24.3(128,517,572–143,689,390) × 2 hmz 10q24.2q25.1(101,324,364–109,677,582) × 2 hmz 11q24.2q25(123,916,776–132,360,867) × 2 hmz 16q11.2q12.2(46,504,466–55,451,871) × 2 hmz 19q13.12q13.32(36,345,999–45,532,009) × 2 hmz	46.4 Mb (2.89%)	Term birth, normal development
65	18	Adverse pregnancy history	46, XY	3p26.1p24.1(8,494,626–26,413,121) × 2 hmz	17.9 Mb	Term birth, normal development
66	18 +	High risk of trisomy 21 screening	46, XX	4q32.1q34.3(161,662,054–181,126,952) × 2 hmz	19.5 Mb	Term birth, normal development
67	19 +	AMA, RSA	46, XY	15q21.1q24.2(49,174,353–76,415,329) × 2 hmz	27.2 Mb	Term birth, normal development
68	30 =	Fetal ventriculomegaly (1.4 cm)	46, XX	1q25.2q31.3(179,562,791–198,041,374) × 2 hmz 1p33p32.3(49,189,774–53,588,443) × 2 hmz 1p31.1p22.1(83,490,160–94,219,311) × 2 hmz 3q22.2q22.3(135,242,870–138,329,862) × 2 hmz 3p14.1p13(65,159,530–71,254,193) × 2 hmz 3q12.1q12.3(99,386,363–102,630,046) × 2 hmz 4q34.3q35.1(178,198,740–183,532,267) × 2 hmz 5q33.3q34(157,473,329–162,124,378) × 2 hmz 8p21.2p12(25,313,218–29,802,727) × 2 hmz 9q22.1q31.2(90,844,062–108,221,369) × 2 hmz 10q21.1q22.1(54,568,807–72,273,380) × 2 hmz 11p11.2p11.12(45,959,522–51,550,787) × 2 hmz 13q31.3q32.3(93,816,292–101,526,284) × 2 hmz 18q11.1q11.2(18,552,516–23,353,126) × 2 hmz 18p11.31p11.21(4,951,983–15,079,294) × 2 hmz 20p13p12.3(61,794–5,436,062) × 2 hmz	129.19 Mb (4.65%)	Term birth, hydrocephalus, abnormal fingers on both hands, learning disability
69	23	Fetal cleft palate	46, XY	8q24.22q24.3(134,714,740–146,292,734) × 2 hmz	11.6 Mb	TOP
70	18 +	AMA	46, XX	10q24.32q25.3(104,021,108–115,579,812) × 2 hmz	11.5 Mb	Term birth, normal development
71	19 +	AMA	46, XY	1p36.12p34.2(22,992,252–40,994,050) × 2 hmz	18 Mb	Term birth, normal development
72	23 +	Hydramnios, AMA	46, XX	2q31.1q32.2(177,156,393–190,026,211) × 2 hmz	12.8 Mb	Preterm birth, normal development
73	20 +	High risk of trisomy 21 screening	46, XX	15q14q21.1(37,475,111–48,299,651) × 2 hmz	10.8 Mb	Term birth, normal development
74	22 +	Hyperechoic nodules in fetal liver	46, XX	2q11.1q12.3(95,550,957–108,770,463) × 2 hmz	13.2 Mb	Term birth, normal development
75	20	AMA, embryo arrest in one of the twin pregnancy	46, XY	5q11.1q13.2(49560858_68826246) × 2 hmz 5p13.2p11(38119461_46383335) × 2 hmz 10q11.22q21.1(48654362_59013629) × 2 hmz 14q32.2q32.33(98824485_107285437) × 2 hmz 17q11.1q11.2(25309336_30880382) × 2 hmz 17p12p11.1(13895964_22217883) × 2 hmz	19.26 Mb 8.26 Mb 10.36 Mb 8.46 Mb 5.57 Mb 8.32 Mb	Term birth, normal development
76	18 +	AMA	46, XY	10q22.3q23.33(79424943_95211586) × 2 hmz	15.79 Mb	Term birth, normal development
77	27	Small fetal BPD for gestation age, mild tricuspid regurgitation	46, XX	6q22.31q23.3(124043730_137024585) × 2 hmz	12.98 Mb	Term birth, normal development
78	19	AMA	47, XX, + 21	arr (21) × 3 18p11.32p11.21(136,304–15,079,294) × 2 hmz	14.9 Mb	TOP
79	21 +	Thickened NT (4.7 mm)	46, XY	3p12.3p11.1(78796314_90485635) × 2 hmz	11.7 Mb	Term birth, normal development
80	19 +	Pregnant women with mental retardation, night blindness, consanguineous marriage	46, XY	13q13.3q21.33(38088920_71004437) × 2 hmz	32.9 Mb	Term birth, normal development
81	25 +	Fetal lung cystic adenoma		5q31.3q34(141922621_162299719) × 2 hmz	20 Mb	TOP
82	22	Fetal enhanced intestinal echo	46, XX	1q32.2q44(208165416_245084139) × 2 hmz	36.9 Mb	Term birth, normal development
83	18 +	High risk of trisomy 1 and trisomy 8 detected by NIPT	46, XY	10q23.1q25.1(87343533_109730397) × 2 hmz	22.4 Mb	Term birth, normal development
84	26	Fetal slightly thickened pulmonary valve, strephenopodia, pulmonary valve and tricuspid valve mild regurgitation	46, XY	3p26.2p25.1(2886527_13828221) × 2 hmz 4p16.3p15.33(3473602_14373371) × 2 hmz 5p13.3p11(31554333_46313469) × 2 hmz	10.9 Mb 10.9 Mb 14.8 Mb	TOP
85	19	Absent fetal nasal bone, high risk of trisomy 18	46, XX	2p16.1p13.2(55018895_72337985) × 2 hmz 2q11.2q31.1(101324333_177929684) × 2 hmz 3q13.33q21.3(119592046_128125154) × 2 hmz 4q34.3q35.2(181657468_190921709) × 2 hmz 4q26q31.21(116436130_145124024) × 2 hmz 5q23.3q33.3(128032159_157973399) × 2 hmz 11q11q13.3(54827208_69837254) × 2 hmz 11p14.3p11.12(21783630_51550787) × 2 hmz 12q12q24.31(46091467_124915560) × 2 hmz 15q22.31q24.1(66572692_73290903) × 2 hmz 17p13.3p13.1(1365961_8388179) × 2 hmz 17q22q24.1(55220296_64161582) × 2 hmz 17q24.3q25.3(69423334_81041760) × 2 hmz 21q22.12q22.3(36678533_48061211) × 2 hmz	339.6 Mb (12.2%)	Term birth, normal development
86	18	hypoplastic nasal bone, high risk of trisomy 21 detected by NIPT	46, XY	3q12.1q13.2(99160747_111326722) × 2 hmz	12.2 Mb	Term birth, normal development
87	18 +	The husband of the pregnant woman carries 46, XY, t (2; 6) (p21; q21)	46, XX	11p15.5p15.4(230751_5408252) × 2 hmz	5.18 Mb	Term birth, normal development
88	30	FGR	46, XY	3q11.2q13.13(97766775_109823273) × 2 hmz 4q27q32.1(121203882_156320512) × 2 hmz	12 Mb 35 Mb	Preterm birth, normal development
89	19 +	Deletion in 1p36.3 detected by NIPT, AMA	46, XX	1p36.33p36.13(888659_18328851) × 2 hmz	17.4 Mb	Term birth
90	20	Thickened NT (3.2 mm)	46, XY	2q24.3q31.1(164542492_174491805) × 2 hmz	10 Mb	Term birth, normal development
91	25 +	Fetal portal-body venous shunt outside the liver	46, XY	4p15.2p11(22224153_49063479) × 2 hmz	26.8 Mb	Term birth, normal development
92	19 +	High risk of trisomy 21 screening	46, XX	8q13.3q21.13(72567811_83036411) × 2 hmz	10.5 Mb	Term birth, normal development
93	18 +	Deep notch a-wave of ductus venosus, adverse pregnancy history	46, XY	13q14.2q21.2(47320304_61602456) × 2 hmz	14.3 Mb	Term birth, normal development
94	18	Adverse pregnancy history	46, XY	2q11.1q11.2(95550958_100799003) × 2 hmz 9p24.1p13.2(8235898_36732597) × 2 hmz 18q11.2q22.1(23795473_66441448) × 2 hmz 20q13.12q13.32(43817586_56736674) × 2 hmz	89.3 Mb (3.21%)	Term birth, normal development
95	18 +	High risk of trisomy 21 screening	46, XY	18q12.2q21.1(35074699_45621145) × 2 hmz	10.55 Mb	Term birth, normal development
96	26	Fetal right ventricle is slightly smaller than the left ventricle, slightly smaller the inner diameter of the pulmonary artery and left and right pulmonary artery, and mild tricuspid regurgitation, high risk of trisomy 21 screening	mos 47, XY, + 22[3] /46, XY [58]	arr (22) × 2.3 (30% mos) 22q12.3q13.33(36679058_51157531) × 2 hmz	14.5 Mb	TOP
97	19 +	High risk of fetal sex chromosome aneuploidy detected by NIPT, AMA	46, XX	3q13.13q22.2(110489883_134438659) × 2 hmz	23.9 Mb	Term birth, normal development
98	20	Fetal bilateral choroid plexus cysts, intracardiac echogenic focus, high risk of fetal sex chromosome aneuploidy detected by NIPT	46, XY	3p13p11.1(72195690_90485635) × 2 hmz 3q11.1q12.2(93558926_100589330) × 2 hmz 4q31.21q32.3(145585354_165933062) × 2 hmz 6q26q27(161702754_170896644) × 2 hmz 11p13p11.12(36146925_51550787) × 2 hmz 11q11q12.2(54827208_60193880) × 2 hmz	75.6 Mb (2.72%)	Term birth, normal development
99	19 +	The pregnant woman carries 46, XX, t (12; 21) (q12; q22)	46, XX, t (12;21) (q12; q22.2) mat	3p12.3q12.2(78304909_100613493) × 2 hmz	22 Mb	Term birth, normal development
100	20 +	Fetal separation of right renal pelvis, intracardiac echogenic focus	46, XY	14q23.2q24.3(62101707_77219310) × 2 hmz	15 Mb	Term birth, normal development
101	28	Fetal multiple calcification foci in fetal abdominal cavity	46, XY	2q24.1q24.3(156,461,811–165,665,567) × 2 hmz 2p13.2p11.2(72,170,192–83,714,557) × 2 hmz	9.2 Mb 20.7 Mb	Term birth, normal development
102	31	Fetal agenesis of the corpus callosum	46, XX	13q21.2q31.1(61,365,983–85,033,012) × 2 hmz	23 Mb	TOP
103	25	FGR	46, XX	6q12q14.1(69812646_82725168) × 2 hmz	12.9 Mb	Preterm birth, normal development
104	30 +	FGR	46, XY, 15ph	6p12.3q13(47138118_74152240) × 2.77 6p25.3p12.3(203877_47207081) × 2 hmz 6q13q27(74056154_170896644) × 2 hmz	27.0 Mb 96.8 Mb	TOP
105	29 +	FGR, increased intestinal echo	46, XY	12p13.2p12.1(10143600_22685434) × 2 hmz	12.5 Mb	Term birth, normal development
106	21	AMA	46, X, inv(Y) (p11.2q11.2) mos 45%	arr (13) × 2 mos hmz (45%)		Term birth, epilepsy, mild mental retardation

*The percentage is calculated by the sum of the size (Mb) of the LOH segments over 5 Mb divided by ~ 2781 Mb.

^**§**^Each case was classified based on its most important invasive diagnostic indication. The classification of indications, arranged in the order of importance are as follows: ultrasound anomalies, positive NIPT results, high risk for Down’ screening, parental abnormal karyotype, consanguinity, previous adverse pregnancies, AMA, suspected fetal infection, and others.

*AMA*, advanced maternal age; *BPD*, biparietal diameter; *dn*, de novo; *FGR*, fetal growth restriction; *hmz*, homozygosity; *NIPT*, noninvasive prenatal testing; *NT*, nuchal translucency; *RSA*, recurrent spontaneous abortion; *SNP*, single nucleotide polymorphism; *TOP*, termination of pregnancy.

### Fetal LOH detected by SNP array

**Figure 1:**
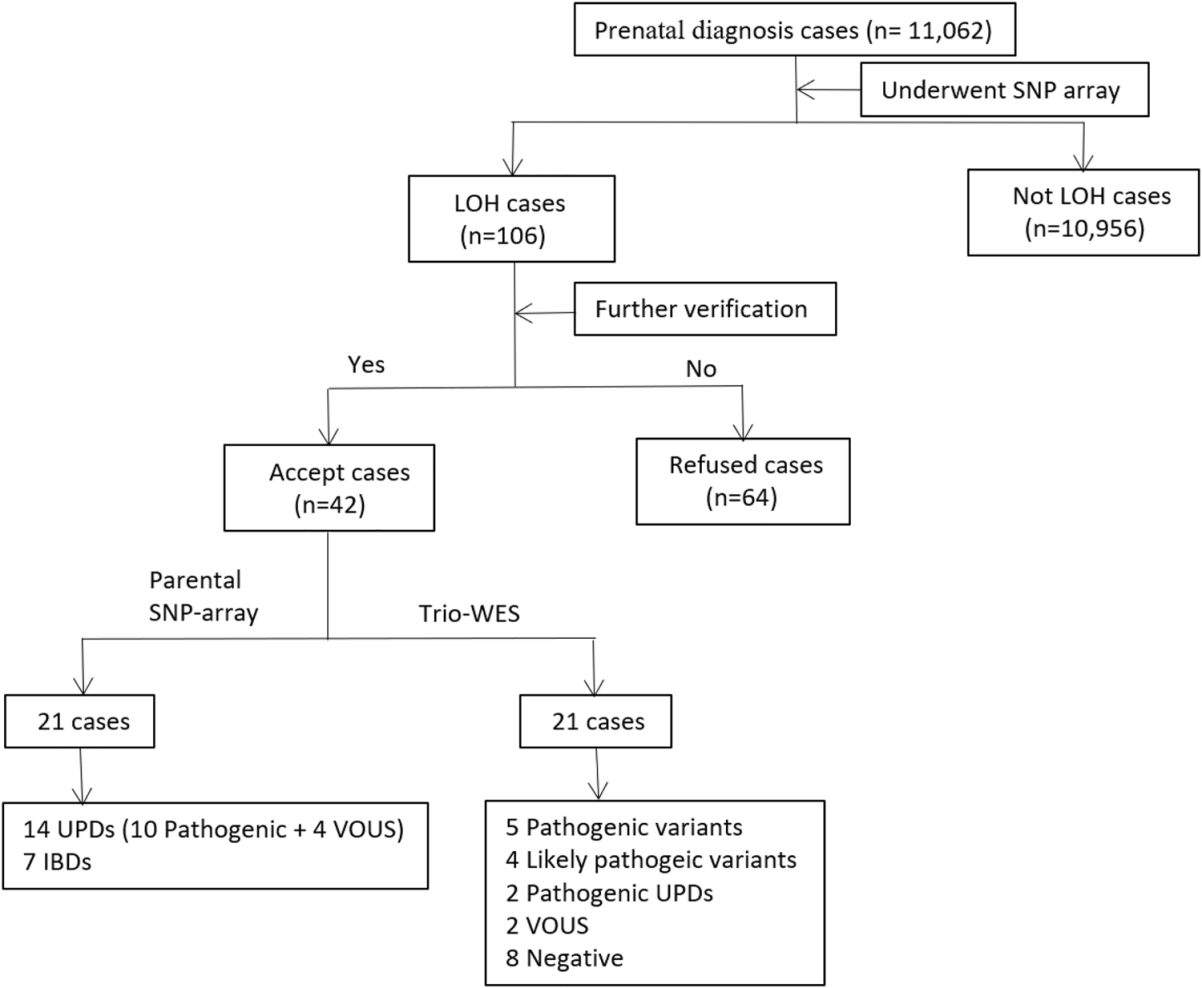
The flow chart of fetal LOH analysis in our cohort. IBD, identity by descent; LOH, loss of heterozygosity; SNP array, single nucleotide polymorphism array; UPD, uniparental disomy; VOUS, variant of unknown significance; WES, whole exome sequencing.

The overall flow of fetal LOH analysis is illustrated in Fig. 1. The detection rate of fetuses with LOH was 0.96% (106/11,062). In 88 (83.0%) fetuses, LOH occurred on a single chromosome, whereas in 18 (17.0%) fetuses, multiple LOHs were detected on different chromosomes. Of the 18 cases with multiple LOHs, two cases (Cases 1 and 64) were confirmed from consanguineous couples, and the remaining 16 cases denied consanguineous marriage.

In single-chromosome LOH, the most frequently involved chromosomes were chromosomes 6 (11.4% (10/88)), 3 (10.2% (9/88)), and 5 (8.0% (7/88)), followed by chromosomes 15 (6.8% (6/88)), 2 (6.8% (6/88)), 8 (6.8% (6/88)), and 13 (6.8% (6/88)) (Tables 2 and 3). LOH involved almost the entire chromosome in seven cases, and the involved chromosomes were chromosomes 2, 6, 13, 14, 18, and 22. In other cases, LOH occurred in only part of the chromosome, ranging from 5.18 to 96.8 Mb. In total, four cases of mosaic LOH were identified, including chromosomes 13 and 14, 5q11.1q35.3, and 7q21.13q36.3, respectively. The mosaicism rate ranged from 30 to 80%.

Both cases (Cases 22 and 28) with LOH on the entire chromosome 6 presented with fetal growth restriction (FGR), and were further diagnosed with paternal and maternal UPD6, respectively. Both patients elected termination of pregnancy (TOP) (Table 2). Isolated segmental LOH was identified on chromosome 6 in 10 cases, of which nine presented with UAs, including FGR, thickened nuchal translucency (NT), enhanced bowel echo, intracardiac echogenic focus, increased umbilical artery resistance index, oligohydramnios, cervical lymphatic hygroma, mild regurgitation of tricuspid valve, fetal bilateral renal enlargement, increased renal echogenicity, reverse a-wave of ductus venosus, and enhanced intestinal echo, resulting in TOP (*n* = 6) and preterm birth (*n* = 1). The other three cases had a favorable outcome.

Isolated LOH on chromosome 3 was identified in nine cases, of which only two presented with UAs, including thickened NT, resulting in spontaneous abortion (*n* = 1). The other seven cases showed no anomalies on prenatal ultrasound and had no obvious abnormal phenotypes after birth.

Isolated fetal LOH on chromosome 5 was identified in seven cases, of which three had abnormal ultrasound findings, including FGR; small fetal head circumference (HC) for gestational age; lethal bone dysplasia (osteogenesis imperfecta type II); micrognathia; small biparietal diameter (BPD), HC, and femur length (FL) for gestational age; bilateral femoral curvature; less than the normal predictive value -2SD; hydrops fetalis; fetal giant bladder; and fetal lung cystic adenoma, resulting in TOP (n = 3) or preterm birth (n = 1), and missed abortion (n = 2); the other case (Case 34) showed minor abnormal phenotypes on prenatal ultrasound, and presented micrognathia after birth.

### UAs in fetal LOH detected by SNP array

In total, 66 fetuses presented UAs, including 22 (33.3%) with structural abnormalities, 24 (36.4%) with ultrasonic soft marker, 18 (27.3%) with FGR and nine (13.6%) with other presentations. The most common soft marker anomaly was thickened NT. The most frequent ultrasonic structural anomalies were cardiovascular (9.1%), skeletal (6.1%), and genitourinary malformations (6.1%). Fetal LOH mostly involved chromosomes 5, 6, and 1 in the group with UAs, and chromosomes 3, 10, and 18 in the group without UAs (Tables 2 and 3).

FGR was detected prenatally in 18 fetuses, of which 11 had FGR as an isolated ultrasound finding. The most frequently involved chromosome was chromosome 6 (n = 6) in cases with FGR, followed by chromosomes 7 (n = 3) and 17 (n = 3). The outcomes of these 18 fetuses included TOP (n = 11), preterm birth (n = 3), and term birth (n = 4). The incidence of TOP was significantly higher in fetuses with FGR than in those without FGR (61.1% (11/18) versus 21.6% (19/88),* p* < 0.01) (Tables 2 and 3).

### UPD results

**Figure 2:**
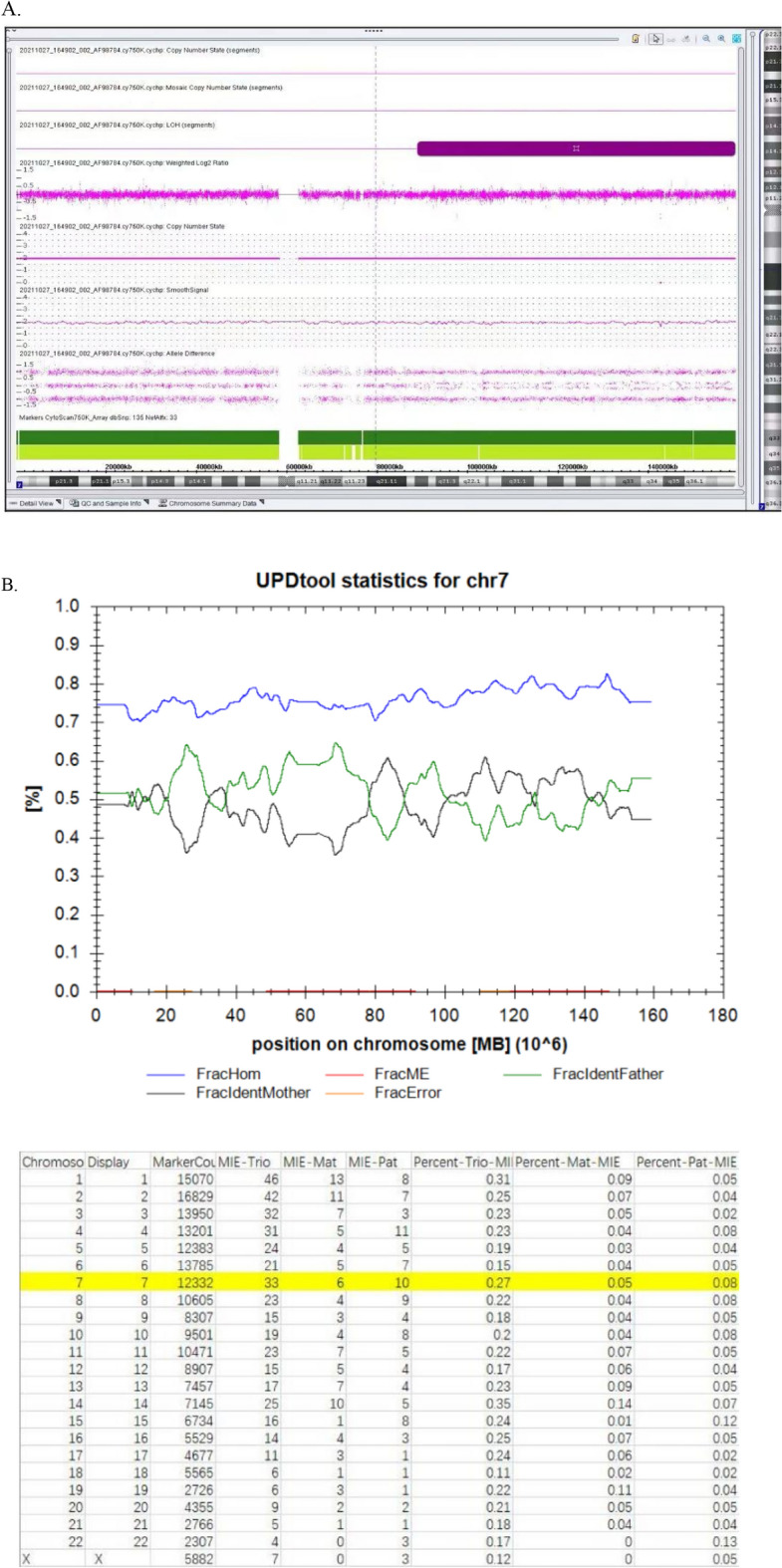

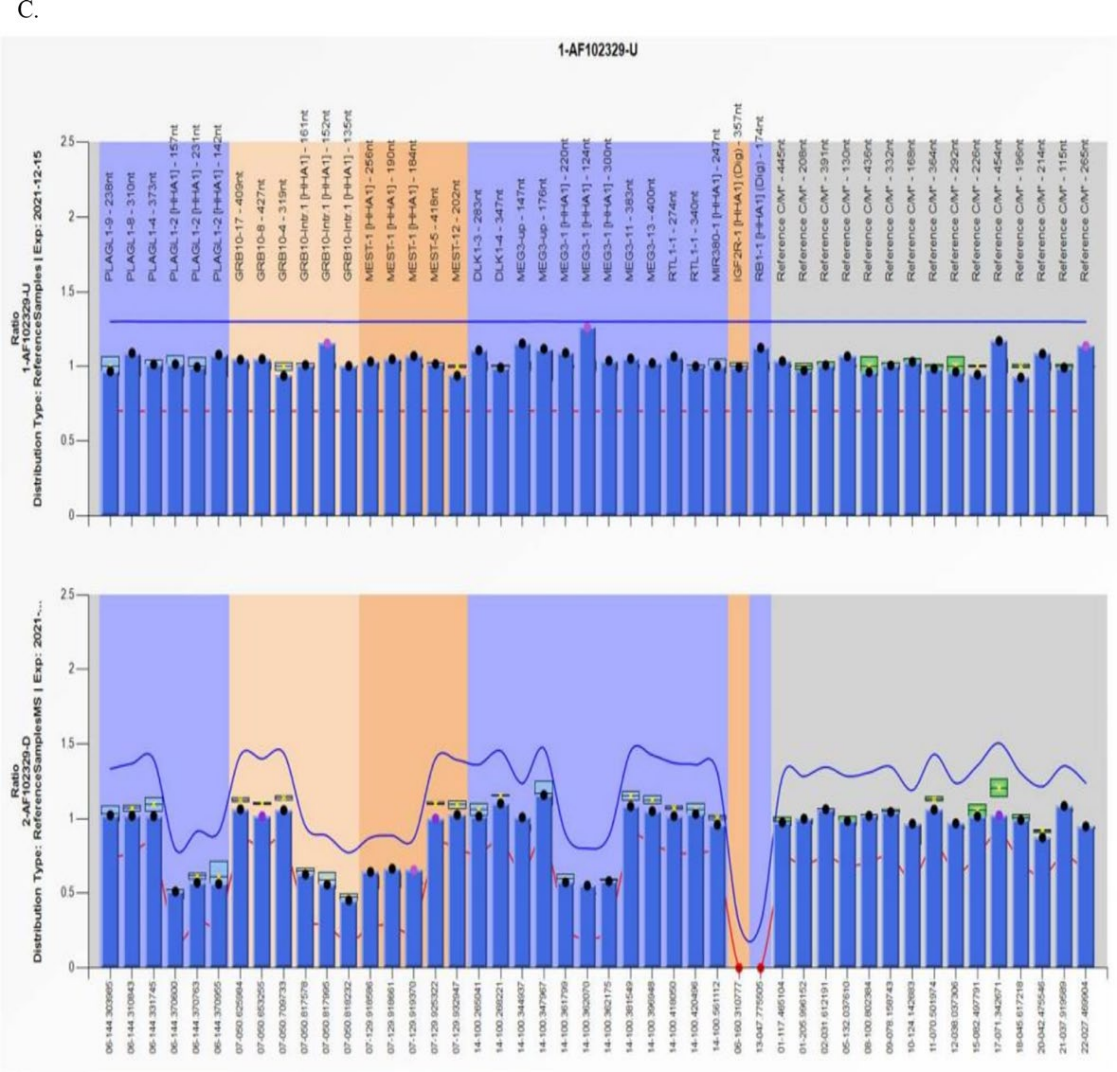
(**A**) 70.4 Mb loss of heterozygosity (LOH) was detected by SNP array (**A**), and further confirmed as maternal uniparental disomy 7 (UPD7) (**B** and **C**) for case 14. Detection of a 70.4 Mb segmental LOH on 7q21.13q36.3 using a SNP array. Purple bars represent stretches of LOH occurring on 7q21.13q36.3. The allele difference panel indicates the genotype for each SNP. Confirmation of low proportion mosaic maternal UPD7 using the methylation-specific multiplex ligation dependent probe amplification (MS-MLPA). MS-MLPA revealed that the relative copy numbers of the three methylation probes in *MEST* gene (maternal methylation gene region, paternal methylation was preferentially expressed) were 0.66, 0.66 and 0.64, respectively, suggesting a possible low proportion mosaic UPD (7) mat. However, the experimental results are near the threshold range, so it cannot be interpreted accurately.

To verify the parental source of fetal LOH, 21 cases of fetal LOH were confirmed by parental SNP array analysis, of which two (9.5%) had paternal UPD (Cases 4 and 22) and 12 (57.1%) had maternal UPD (Cases 4 (UPD 14 pat), 6 (UPD7 mat), 14 (mosaic UPD7 mat), 19 (UPD15 mat), 21 (UPD 16 mat), 22 (UPD6 pat), 24 (UPD15 mat), 25 (UPD11 mat), 26 (UPD7p22.3p12.2 mat), and 30 (UPD11 mat)); four cases were confirmed to have IBD (Cases 36–42). Among the 14 cases with UPD, Cases 19 and 24 were diagnosed with Prader–Willi syndrome (PWS), Cases 6, 14, 25, 26, and 30 were determined to have Silver-Russell syndrome (SRS), and Cases 4 and 22 were diagnosed with Kagami-Ogata syndrome (KOS) and transient neonatal diabetes mellitus, respectively. No confirmed imprinted genes were detected in Cases 20, 23, 27, and 28, and UPD16 mat was found in Case 21, thus classifying them as variants of uncertain significance (VOUS). Among the 14 UPDs, notably, amniocentesis was performed in Case 14, due to a fetal right aortic arch with aberrant left subclavian artery detected on ultrasound, and LOH with a size of 70.4 Mb was observed in 7q21.13q36.3 by SNP array (Fig. 2A). First, parental SNP array verification indicated that it was not possible to determine whether the source of the LOH on chromosome 7 was paternal or maternal (Fig. 2B); then, MS-MLPA for the methylation analysis of 7q21.13q3615 loci revealed that the relative copy numbers of the three methylation probes (184, 190, and 256 bp) in the *MEST* gene (maternal methylation gene region, paternal methylation was preferentially expressed) were 0.66, 0.66, and 0.64, respectively, suggesting a possible low proportion mosaic maternal UPD7 associated with Silver-Russell syndrome (Fig. 2C). However, the experimental result was close to the threshold range; thus, it could not be determined and interpreted accurately. The fetus was delivered at term and had feeding difficulties after birth, whereas three months after delivery, the child's height and development were normal, except for light weight (Table 2).

Twenty-one cases of fetal LOH were further verified by trio-WES, of which one (4.8%) had paternal UPD and four (19.0%) had maternal UPD. Notably, trio-WES was performed in Case 12, confirming maternal UPD15 associated with PWS, and the pregnancy was terminated at 28 weeks (Table 2). No definite imprinted genes were observed in the other four UPDs, and these were classified as VOUS.

### Karyotyping results

Karyotyping was performed successfully in all 106 fetuses with LOH. In total, 97 cases yielded normal results, seven cases had abnormal karyotyping results, including 47,XX, + mar dn, 46,X,i(Xq)[40]/45,X[14], 46,XX,t(4;5)(q31;q23)pat, 46,XY,t(11;22)(q24.1;q12.3)pat, 46,XX,t(12;21)(q12;q22.2)mat, 47,XX, + 21, and mos 47,XY, + 22[3]/46,XY[58], and 2 cases had normal polymorphic variation (46, XY, 15ph, and 46,X,inv(Y)(p11.2q11.2) mos 45%, respectively).

### Gene mutation results

Trio-WES was performed in 21 cases to detect gene mutation of autosomal recessive diseases in addition to UPD, and 11 results were clinically significant, including five pathogenic variants, four likely pathogenic variants, and two pathogenic UPDs. Among these clinically significant results, trio-WES identified homozygous mutations in autosomal recessive diseases attributed to LOHs in three cases (Cases 1, 9, and 15).

**Figure 3:**
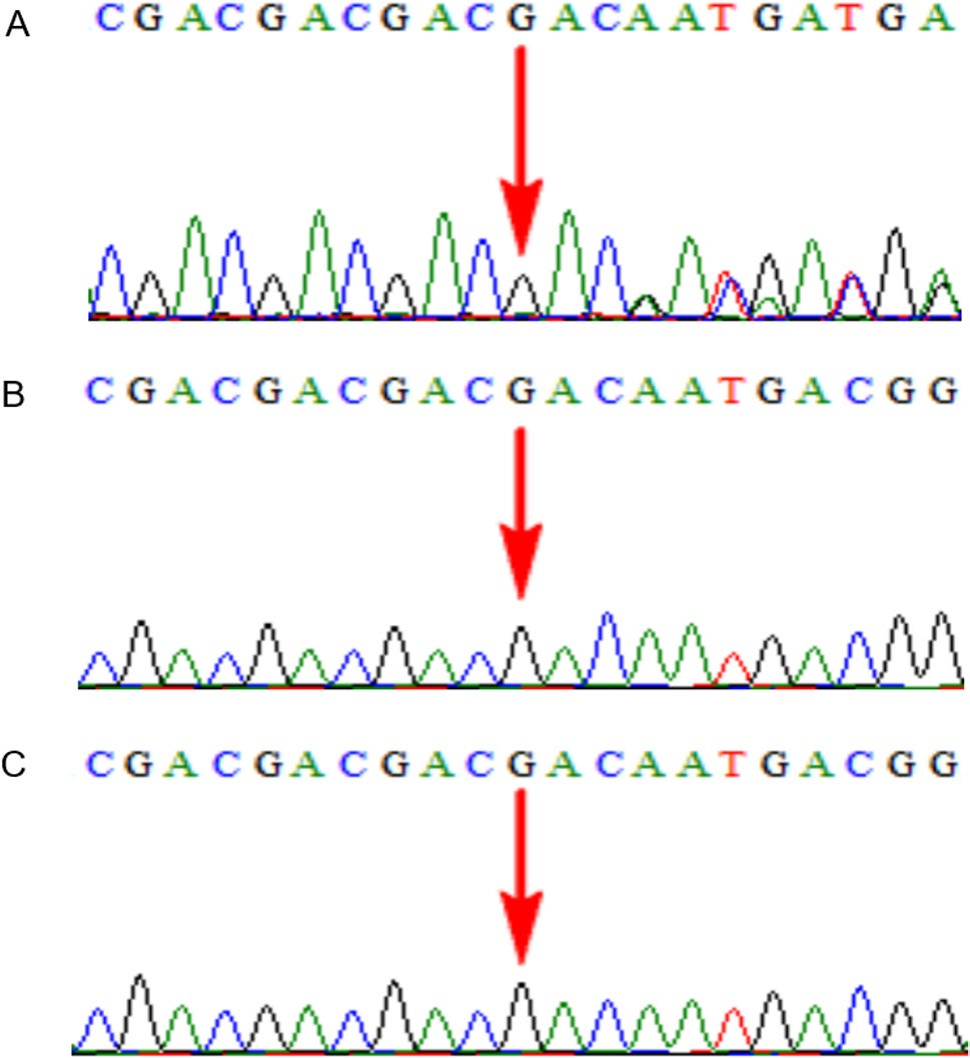
Trio whole exome sequencing results for case 15. The female infant harbored a de novo heterozygous mutation, NM _000095: c.1417_1419dup(p.D473dup), in *COMP* in the female infant. (**A**) The female infant; (**B**) The mother; (**C**) The father.

**Figure 4:**
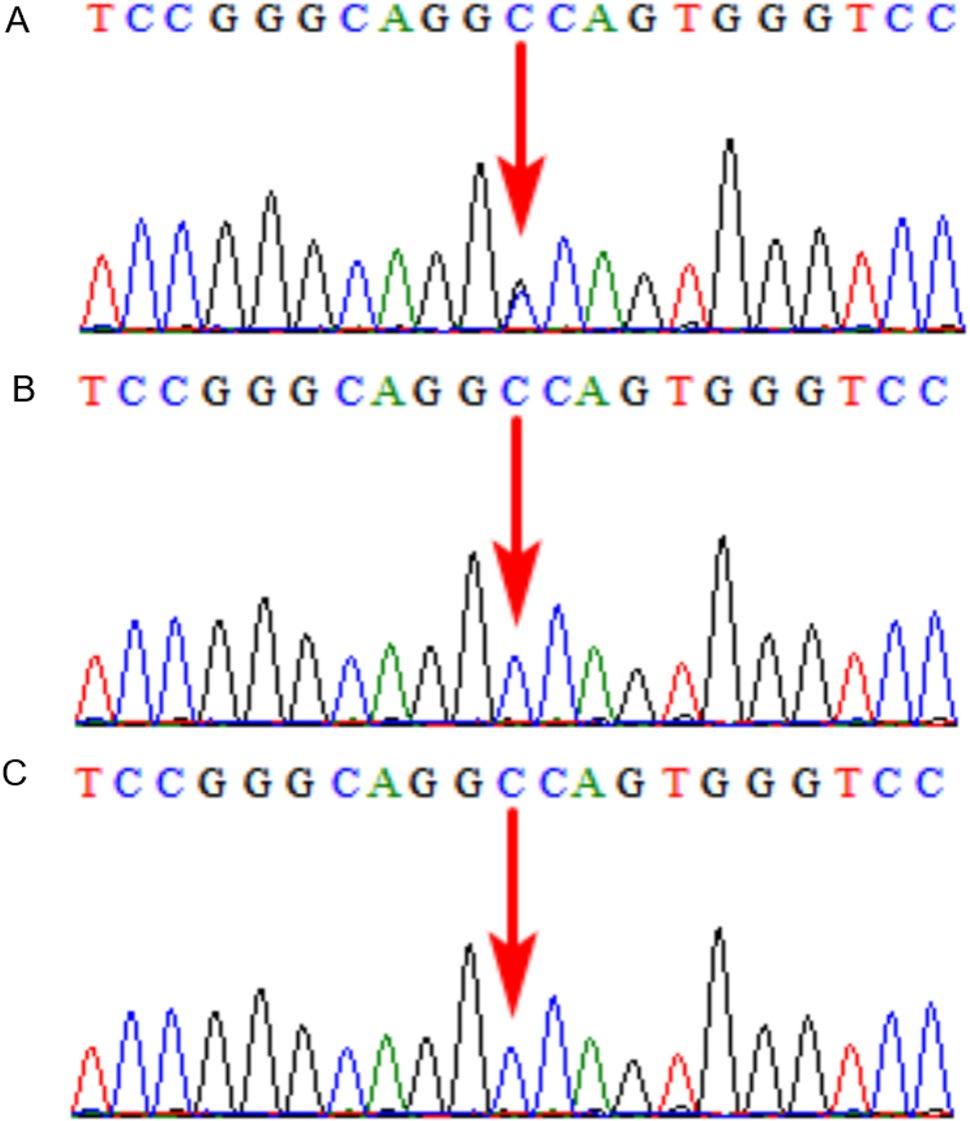
Trio whole exome sequencing results for case 18. The fetus harbored a de novo missense variant, NM_000088: c.1436G > C p.G479A, in *COL1A1* on chromosome 17. (**A**) The fetus; (**B**) The mother; (**C**) The father.

**Figure 5:**
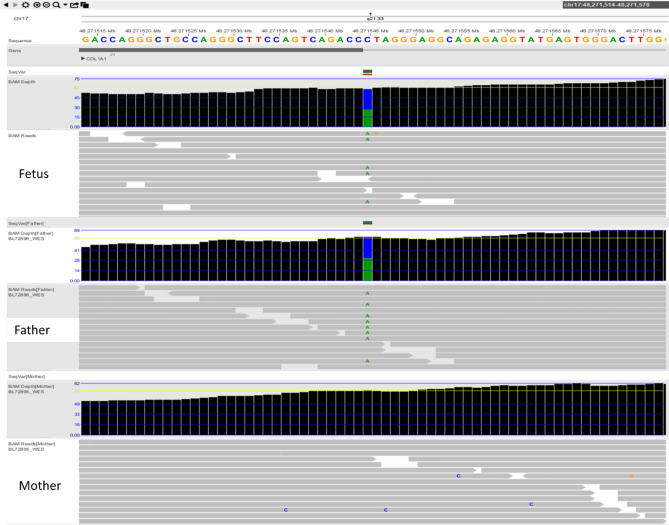
Trio whole-exome sequencing results for case 29. The fetus harbored an inherited paternally splicing variant, NM_000088.4: c.1615-1G > T (p. G802V), in *COL1A1* on chromosome 17.

In Case 11, fetal BPD was small for gestational age as detected by ultrasound, SNP array showed a 38 Mb LOH in 14q13.1q24.2, prenatal trio-WES was declined, and the fetus was delivered at term. At five months old, the infant was 60 cm tall, and often arched her back; brain MRI at three months of age revealed asymmetrical bilateral ventricles, the left lateral ventricle was larger than the right, and some extracerebral spaces were slightly widened. Low T1W1 and high T2W1 signals were observed in the bilateral maxillary, ethmoid, and sphenoid sinuses. Postnatal trio-WES ruled out UPD14, and showed a de novo heterozygous mutation, NM_000095: c.1417_1419dup (p. D473dup), in *COMP* in the female infant (Fig. 3), which was an incidental finding associated with autosomal dominant pesudoachondroplasia (PSACH, OMIM:177,170), multiple epiphyseal dysplasia 1 (EDM1, OMIM:132,400), and carpal tunnel syndrome 2 (CTS2, OMIM:619,161). In Case 18, amniocentesis was performed, as the fetal left femur was slightly curved, and the SNP array revealed a 20 Mb LOH located in 5q23.2q32. Prenatal trio-WES indicated a de novo missense variant, NM_000088: c.1436G > C p.G479A , in *COL1A1* (120,150) on chromosome 17 in the fetus (Fig. 4), associated with osteogenesis imperfecta, type I (OMIM:166,200), type II (OMIM:166,210), type III (OMIM:259,420), type IV (OMIM:166,220), Ehlers-Danlos syndrome, arthrochalasia type 1 (OMIM:130,060), Caffey disease (OMIM:114,000), and bone mineral density variation QTL, steoporosis (OMIM:166,710); the couple elected TOP at 26 weeks. In Case 29, amniocentesis was performed, as the fetal bilateral femoral curvature was less than the normal predictive value -2SD on ultrasound, and the SNP array revealed a 46.1 Mb LOH located in 5p15.33p11. Prenatal trio-WES indicated an inherited paternally splicing variant, NM_000088.4: c.1615-1G > T (p.G802V), in *COL1A1* (120,150) on chromosome 17 in the fetus (Fig. 5), associated with autosomal dominant osteogenesis imperfecta, and the pregnancy was terminated at 23 weeks (Table 2).

### Perinatal outcome and follow-up

The pregnancy outcomes of the 106 fetuses with LOH were as follows: 67 term births (of which two resulted in neonatal death, and one had an abnormal phenotype after birth), 29 TOPs, three preterm births, two fetal deaths, and five miscarriages. A full-term infant (Case 14) was diagnosed with feeding difficulties after birth, and three months after delivery, the child's height and development were normal, except for low weight. A preterm infant (Case 2) was diagnosed postnatally with autism. One fetus (Case 11) showed fetal BPD that was small for gestation age on prenatal ultrasound screening and was delivered vaginally at term. The infant showed growth delay (60 cm tall at five months old), hypotonia, and often arched her back. The rate of adverse pregnancy outcomes in fetuses with LOH with UAs was 52.4% (22/42), and 32.8% (21/64) in those that did not show positive ultrasound findings (*p* < 0.05).

In 42 fetuses with LOH accepted further genetic analysis, the pregnancy outcomes were as follows: 24 term births (of which two resulted in neonatal death (Case 1 and 5), and three had abnormal phenotypes after birth (Case 11 was diagnosed with short stature, and often arches her back, Case 14 had feeding difficulties, and Case 34 presented with micrognathia), 18 TOPs, and one preterm birth (Case 2 had hand deformity and autism). In 64 fetuses with LOH declined further genetic analysis, the pregnancy outcomes were as follows: 42 term births (two had abnormal phenotypes after birth (Case 68 was diagnosed with hydrocephalus, abnormal fingers on both hands, learning disability, and Case 106 presented with epilepsy, mild mental retardation), 12 TOPs, five missed abortions, four preterm births, and one fetal demise in utero. The rate of the adverse pregnancy outcomes in fetal LOH accepted further genetic analysis was 54.8% (23/42), and 37.5% (24/64) in those declined further genetic analysis (*p* < 0.05).

## Discussion

In our cohort, we investigated the clinical significance of fetal LOH as well as the correlation between fetal LOH and its clinical features. The detection rate of LOH meeting the reporting threshold in our study was 0.96% (106/11,062), slightly lower than the 0.97% (100/10,294) reported by Liu et al^14^., but significantly higher than the 0.43% (22/5063) reported by Liang et al^15^., the difference might be attributed to different microarray platforms, the size threshold of LOH reported, sample sizes and the type of the specimen. The threshold in our cohort was set according to that reported by Rehder et al^9^. and Hoppman et al^16^. In addition, none of the clinically significant LOHs occurring in chromosome X were reported due to lack of adverse family history associated with X-linked disorders. When fetal LOH occurred on a single chromosome, chromosomes 6 and 5 were the most commonly involved; whereas Liu et al^14^. showed that LOH was more likely to occur in chromosomes X, 2, and 16. The discordance may be due to the array platform and the reporting threshold of LOH studied.

Clinically significant imprinting disorders should be valued, especially for UPD involving imprinted chromosomes 6, 7, 11, 14, 15, and 20^17,18^. The clinical significance of UPD is closely associated with the affected imprinted region and genes in addition to parental origin^15,19^. Furthermore, it is unclear whether imprinting affects UPD 16^20–22^, since the outcomes of the carriers were variable, from normal growth to delayed growth^22^. Notably, in Cases 21 and 27, pregnancies with confirmed maternal UPD16 were terminated owing to UAs and abnormal genetic results.

In our cohort, 62.3% (66/106) of fetuses with LOH presented UAs, the most common UA was FGR or FGR combined with other indications (18/66 (27.3%)), and the most frequently observed ultrasound structure anomalies were cardiovascular system (6/66 (9.1%)), skeletal (4/66 (6.1%)), and genitourinary malformations (4/66 (6.1%)). Seventeen percent (18/106) of fetuses with LOH had FGR, UPDs 2, 7, 14, 15, and 16 were the underlying genetic causes of FGR^17,23,24^. The possible pathogenesis encompasses homozygous pathogenic variants in single gene diseases, imprinting effects, or confined placental mosaicism (CPM)^25^. Thus, UPD is one possible genetic factor resulting in FGR. Monitoring fetal growth via ultrasound is essential for the management of fetal LOH. Indicative prenatal ultrasound findings can be observed in patients with Beckwith-Wiedemann syndrome and SRS^26,27^. In our cohort, four cases (Cases 6, 25, 26, and 30) with LOH showed FGR or FGR combined with other indications, of which four UPDs were confirmed. The genetic causes underlying FGR were maternal UPD7 and UPD11 associated with SRS^17^, and TOP was elected owing to unfavorable outcomes. Patients with UPD14 showed multiple UAs, resulting in unfavorable outcomes^28,29^. Paternal UPD14 associated with KOS was confirmed in Case 4 with polyhydramnios (amniotic fluid index: 38.7 cm), and the pregnancy was terminated. Notably, the rate of adverse pregnancy outcomes in fetuses with LOH and UAs (36.4%) was higher than in those without UAs (15.0%) (*p* < 0.05). Our data demonstrate that regular ultrasound screening is essential to closely monitor the development of fetuses with LOH.

LOH also provides certain signs for investigating homozygous variants in autosomal recessive single gene diseases besides UPD and imprinting effects. In three cases in our cohort, pathogenic homozygous variants in single-gene diseases were further identified via trio-WES, resulting in UAs. Therefore, trio-WES should be first performed for its ability to verify UPD as well as identify homozygous mutations simultaneously (Cases 11, 16, and 17). Furthermore, six cases (Cases 2, 11, 16, 17, 18, and 29) had incidental findings of clinically significant variants. Our study also shows that trio-WES could identify incidental pathogenic mutations in addition to homozygous variants attributed to LOH. Therefore, trio-WES should be recommended first for fetal LOH, especially in fetuses with structural anomalies and/or consanguineous parents.

The study had some limitations. First, although it was a retrospective multicenter study, the sample size was not large enough and the follow-up period was not long enough, so some clinical features may have been missed. Studies with larger populations and longer follow-up will be needed. Second, the parental origin of LOH was further identified in only 39.6% of cases and none of the placental tissue in these cases was further investigated to confirm CPM.

## Conclusion

We explored the clinical significance and features of fetal LOH. Various molecular genetic testing techniques, such as parental SNP array verification, trio-WES, MS-MLPA, regular and systematic ultrasonic examination, and placental study when necessary, should be comprehensively performed to precisely assess the prognosis of fetal LOH and guide the management of the affected pregnancy.

## Data Availability

The data used to support the findings of this study are available from the corresponding author upon request.

## Abbreviations

BPD: Biparietal diameter
CMA: Chromosomal microarray analysis
CNV: Copy number variation
FL: Femur length
FGR: Fetal growth restriction
HC: Head circumference
IBD: Identity by descent
LOH: Loss of heterozygosity
MS-MLPA: Methylation-specific multiplex ligation-dependent probe amplification
NT: Nuchal translucency
PWS: Prader–Willi syndrome
KOS: Kagami-Ogata syndrome
SRS: Silver-Russell syndrome
TOP: Termination of pregnancy
UAs: Ultrasound anomalies
UPD: Uniparental disomy
WES: Whole-exome sequencing
VOUS: Variants of uncertain significance
